# Editorial: Systems biology approach for the mechanisms underlying chronic liver disease

**DOI:** 10.3389/fmed.2022.989976

**Published:** 2022-09-09

**Authors:** Zhengtao Liu, Yafeng Zhu, Cheng Zhang, Sunjae Lee

**Affiliations:** ^1^Shulan International Medical College, Zhejiang Shuren University, Hangzhou, China; ^2^NHC Key Laboratory of Combined Multi-Organ Transplantation, Key Laboratory of the Diagnosis and Treatment of Organ Transplantation, CAMS, First Affiliated Hospital, School of Medicine, Zhejiang University, Hangzhou, China; ^3^Shulan (Hangzhou) Hospital, Hangzhou, China; ^4^Medical Research Center, Sun Yat-sen Memorial Hospital, Sun Yat-sen University, Guangzhou, China; ^5^Science for Life Laboratory, KTH Royal Institute of Technology, Stockholm, Sweden; ^6^School of Life Sciences, Gwangju Institute of Science and Technology, Gwangju, South Korea

**Keywords:** chronic liver disease, omics, systematic biology, integrative analysis, big data

Chronic liver disease (CLD) including chronic hepatitis, alcoholic liver disease (ALD), non-alcoholic fatty liver disease (NAFLD) and liver cirrhosis affects the mortality in general populations. Correspondingly, many therapeutic interventions from anti-viral treatment to liver transplantation are devoted to cure or control CLD developments. Fortunately, the incidence of viral hepatitis decreased rapidly following the wide-spread use of vaccinations and specific medications (ribavirin and interferon) (1). However, concerns were raised for sharp increases on metabolic liver disease like NAFLD and metabolic dysfunction-associated fatty liver disease (MAFLD) (2).

Meanwhile, systematic knowledge of therapeutic means is also crucial to improve the efficiency of CLD therapy. Taking liver transplantation (LT) as an example, the risk profile for prognosis of LT recipients was defined by a network constructed from factors from donor (D), recipient (R), graft, and surgical aspects (3). Currently, growing evidence showed D/R Interactions (mismatch on ABO blood type and graft size, etc.) were also important components for recipient prognosis. Mechanisms for CLD and its treatment are a complex process with potential confounders inside. Therefore, comprehensive knowledge of systematic frames is urgently needed for further CLD prevention and technological development.

Four papers published in this Research Topic are mainly related to Pathogenesis of ALDs, liver fibrosis, biliary atresia, and mesenchymal stem cells (MSCs) therapy for CLDs. Yang et al., reviewed the therapeutic effects of MSCs on CLD therapies. They reexamined the effect of MSCs on improvement of liver regeneration, fibrosis, and the immunomodulatory micro-environment. Meanwhile, factors associated with MSCs differentiation and its safe use in clinical trials were also discussed. As we know, MSCs exerted its therapeutic potentials for liver cirrhosis, and MSCs is a hot topic which is much closer to clinical applications. Yang et al. summarized our current knowledge on MSCs and its clinical potential for CLDs within a systematic frame. Further studies of the connection between MSCs transplantation and clinical outcomes would be worthwhile. Nga et al., mentioned the importance of Interleukin-10 (IL-10) on a potential target for treatment of liver fibrosis based on mouse models. They produced a mouse with severe liver fibrosis *via* interventions of carbon tetrachloride (CCl4) plus thermoneutral (TN) treatment. They found that a medium from brown adipocytes can attenuate the activation of hepatic stellate cells (HSCs) when combined with IL-10. They considered the complexity of liver fibrosis with regard to refractory clinical symptoms. The team of Yi provided potential approaches to reverse liver fibrosis in mice. Further in-depth animal study regarding IL-10 administration might be useful for auxiliary therapy of liver fibrosis. Additionally, Ye et al., reported the associations between frequency of complementarity determining region 3 (CDR3) and biliary atresia (BA) susceptibility. CDR3 is the only non-germline coding region for T-cell receptors (TR) which might be utilized as potent targets for further immuno-therapy in BA patients. Finally, Zuo et al., raised their speculations on the molecular mechanism of miR-182-5p/FOXO1 axis in ALD development. They first built the miRNA-mRNA connections and found the miR-182-5p/FOXO1 as an important regulatory axis based on exploration of omics data from the GEO database. Further validations *in vitro*/*in vivo* also confirmed the role of miR-182-5p/FOXO1 in ALD development and their interactive connections. Finally, authors concluded the up-regulated miR-182-5p caused by alcohol consumption can inhibit the FOXO1 expression which will further cause variations on lipid related genes and triglyceride accumulation in the liver. Indeed, this is an interesting study on ALD mechanisms from exploration of big data to molecular validation in cellular/animal models.

More and more big data (transcriptomics, proteomics, and metabolomics) is deposited in various databases. Usually, only one paper would not fully mine the amount of data inside. It is necessary and worthwhile to encourage re-analysis and combinative analysis on several published data sources. It is preferable for omics data to be open and shared by all scholars without any barriers.

All in all, our Research Topic provided a platform for clinicians to present their most-updated results and opinions on mechanisms and therapy for CLDs. Indeed, the contents in such a Research Topic are insufficient for mechanistic study of CLDs. Further systematic study is needed for better prevention and treatment of CLDs.

## Author contributions

ZL and YZ wrote the manuscript. All authors critically revised the manuscript for important intellectual content.

## Funding

This work was supported by National Natural Science Foundation of China (81902813), Zhejiang Provincial Natural Science Foundation of China (LY22H030008), and CSCO (Chinese Society of Clinical Oncology)-Bayer Tumor Research Funding (Y-bayer202001/zb-0003).

## Conflict of interest

The authors declare that the research was conducted in the absence of any commercial or financial relationships that could be construed as a potential conflict of interest.

## Publisher's note

All claims expressed in this article are solely those of the authors and do not necessarily represent those of their affiliated organizations, or those of the publisher, the editors and the reviewers. Any product that may be evaluated in this article, or claim that may be made by its manufacturer, is not guaranteed or endorsed by the publisher.

## References

[1] Cox AL El-Sayed MH Kao J-H Lazarus JV Lemoine M Lok AS. Progress towards elimination goals for viral hepatitis. Nat Rev Gastroenterol Hepatol. (2020) 17:533–42. 10.1038/s41575-020-0332-6 32704164 PMC7376316

[2] Younossi Z Tacke F Arrese M Chander Sharma B Mostafa I Bugianesi E. Global perspectives on nonalcoholic fatty liver disease and nonalcoholic steatohepatitis. Hepatology. (2019) 69:2672–82. 10.1002/hep.30251 30179269

[3] Liu Z Xu J Que S Geng L Zhou L Mardinoglu A. Recent progress and future direction for the application of multiomics data in clinical liver transplantation. J Clin Translat Hepatol. (2022) 10:363. 10.14218/JCTH.2021.00219 35528975 PMC9039708

